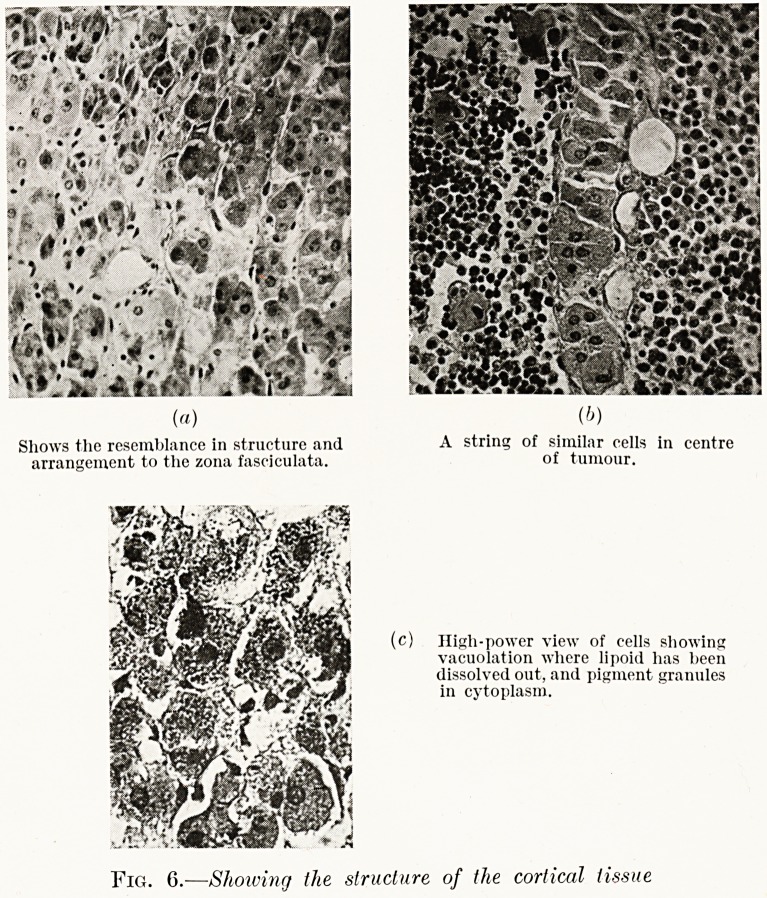# Suprarenal Virilism. With an Account of a Case

**Published:** 1926

**Authors:** Edward Fawcett, Geoffrey Hadfield, Percy Phillips

**Affiliations:** Professor of Anatomy, University of Bristol; Pathologist to General Hospital, Consulting Pathologist to the Southmead Hospital, Bristol; Medical Superintendent of the Southmead Hospital, Bristol


### SUPRARENAL VIRILISM.

### WITH AN ACCOUNT OF A CASE.

BY

EDWARD FAWCETT, M.D., C.M., F.R.S.,
*Professor of Anatomy, University of Bristol,*

GEOFFREY HADFIELD, M.D., M.R.C.P. Lond.,
*Pathologist to General Hospital, Consulting Pathologist to the Southmead Hospital, Bristol,*

AND

PERCY PHILLIPS, M.Sc., M.B., B.Ch. Bristol,
*Medical Superintendent of the Southmead Hospital, Bristol.*

---

### INTRODUCTION.

WE cannot afford to ignore, as physicians or citizens, the biological attributes of sex in the study of behaviour, achievement or disease. The biological sciences, borrowing many facts from medical and pathological experience, have established a few fundamental principles of great value in the study of primary and secondary sex - differentiation, and in the light of these principles our knowledge of disease accompanied by altered sex characters is rapidly enlarging, whilst in others an alteration in the mechanisms which determine sex-differentiation is being recognised as a causative factor.

The case here reported illustrates the part played by the hormone of the suprarenal cortex in the establishment and maintenance of the secondary sex characters, a relation which has been made clear, chiefly by, what is now, the commonplace observation that an excess of functioning suprarenal cortex in girls, women and boys is associated with the development of male secondary characters.

The sexual fate of the individual human being is normally determined at the moment of fertilisation, such primary sex-differentiation being apparently entirely due to the number, variety and arrangement of the chromosomes in the conjugation-nucleus of the ovum. The first trace of sex-differentiation is seen in the gonad, which appears early in embryonic life. Following this differentiation the gonads produce hormones which regulate all subsequent secondary sex characters ; once primary sex-differentiation is accomplished secondary sex-differentiation is handed over entirely to hormonic control. Thus, castration in infancy in either sex leads to the regression of sex characters towards a neuter type, and, if followed by implantation of a gonad of the opposite sex, the characters of the sex of the implanted gonad appear. It thus follows that each sex contains in its cells the factors for the secondary sex characters of the other sex.

Other hormones influence or act with those of the gonads. For instance, under-action of the anterior lobe of the pituitary is associated with diminution in development and function of the genital system, with a tendency in males to assume the female physique. Hypothyroidism may be associated with early maturity in girls ; and excess of the hormone secreted by the cortex of the suprarenal is, in girls and women before the menopause, associated with the development of male secondary characteristics.

This association between overgrowth or tumour formation of the suprarenal cortex and virilism is very striking. No such reactions arise from tumours of the medulla of the gland, which are neuro-blastomata, occur in young male children, and produce extensive metastases, often in the skull bones. The cortical tumours may be congenital, when they are bilateral and hyperplastic ; or acquired, when they are unilateral and neoplastic. Of female pseudo-hermaphrodites 15 per cent. have bilateral cortical hyperplasia. Unilateral new growths (suprarenal hypernephromata) produce alterations in secondary sex characters, almost invariably, in girls, whose voice, physique and hirsuties approximate to the male type, whilst hypertrophy of the clitoris is very common. In women before the menopause half the cases of suprarenal cortical tumour produce virilism. Although similar tumours have been described in men, it is doubtful whether they are ever associated with alterations in secondary sex characters. Yet in boys the association is invariably present, producing the " infant Hercules " type.

There is no sharp line of demarcation between the congenital bilateral hyperplasias of the gland and the acquired unilateral cortical new growths, both of which are so often associated with virilism. The case we describe is almost certainly an example of congenital bilateral hyperplasia of the suprarenal cortex, producing abnormalities in sexual characters of the hormonic, pseudo-hermaphrodite type.

### Case Report.

*History.*—A rather withered old woman of 70 years was admitted to the Southmead Hospital acutely ill from the effects of acute intestinal obstruction. She had a surprisingly deep voice and her face was covered by a stubbly beard of strong black hair, definitely situated in the masculine beard area. She had been ill off and on for several weeks, and it was later found that she had been lately catheterised several times for retention of urine. Considerable abnormality was found in the external genital organs and, although the patient was very reticent, she said she had never menstruated, the abnormality had always been present, and she had had no trouble with micturition, except during the last few weeks. A huge tumour filled the left loin and a smaller mass was felt in the right. These she had not noticed except for a few months before admission. Her symptoms of obstruction were of a few days' duration only. In all respects her physique conformed to the masculine type, the development of the breasts was rudimentary, the shape and development of the bony pelvis and the disposition of fat and pubic hair of the male type. An operation to relieve the obstruction was undertaken immediately, but she died on the following day.

EXTRACTS FROM POST-MORTEM RECORD.

*External Genital Organs.*—The mons veneris and labia majora were small, the nymphæ absent. The clitoris was greatly hypertrophied, 4 in. in length and had a well-marked glans. Its under surface was grooved along its whole length, the groove leading to a female type of urethral opening.

*Internal Genital Organs.*—The uterus was ill-developed and rudimentary. It was 2¼ in. long, and its wall nowhere thicker than ¼ in. The fundus was not developed, the cavity roomy and not constricted in the cervical portion. The arbor vitæ uteri was well marked, and occupied the lower two-thirds of the internal surface. Continuous with the uterus was a thin-walled vagina hardly admitting the forefinger above and becoming conical below, where it opened to the exterior by a small orifice just admitting a probe and lying immediately behind that of the urethra. The broad ligaments were thin, the Fallopian tubes small and fragile, but their fimbriated ends were well formed. A slender round ligament was present. Both ovaries were present but small and fibrotic, and no active ovarian tissue was found in them on section nor any indication that they had ever been the site of ovulation.

*Urinary Organs.*—The clitoris was continuous, with well-formed corpora cavernosa and spongiosa. At the base of the bladder and investing the urethra were two small lateral and one larger mesial mass, which on section had a structure identical with that of the male prostate. The mesial mass projected ¾ in. into a thick-walled bladder, forming a dome-shaped swelling about 1 in. in diameter at its base which was obstructing the internal urethral orifice. Histologically its glandular tissue was hyperplastic, and many tubules showed dilatation and papillary projection of their epithelium similar to the change found in senile hypertrophy in males.

A systematic search was made for testicular tissue; none was found.

### PATHOLOGICAL EXAMINATION OF SUPRARENAL TUMOURS.

The left loin was filled by a large tumour mass, 7½ in. long by 6½ in. broad by 4 in. thick, irregularly lobulated, not unduly adherent and non-infiltrating. Occupying the site of the right suprarenal and partially filling that loin was another large mass of the same naked-eye appearance and consistency, measuring 4½ in. by 3 in. by 3 in., but in shape roughly preserving the form of the normal suprarenal.

Both tumours on section were extremely greasy. The larger showed irregular lobulation and considerable old and recent interstitial hemorrhage. The lobules, roughly ovoid, about 1½ in. long by ¾ in. broad, and devoid of any naked-eye pattern, were separated from each other by broad branching, ashy-grey bands and fasciculi, which formed a wide-meshed network originating in a surface layer of the same tissue constituting an external capsule for the tumour. The lobules were found to be composed of hemorrhagic fat. The capsule and its interlobular branches were composed of tumour tissue.

The capsule of the tumour on the right side was thicker and more uniform, and for a distance of 2 to 3 in. from the lower pole it had the typical colour and naked-eye appearance of normal suprarenal cortex, giving the impression of a suprarenal gland greatly distended by a central new formation over which the cortex was tightly stretched. The kidney below this tumour was normal and not infiltrated. The left kidney showed no naked-eye abnormality.

*Microscopic Examination.*—A large fraction of the bulk of the tumour was composed of hemorrhagic fat, the tumour tissue being confined to the capsule and its interlobular branches. The tumour tissue was well preserved at the surface, but large tracts in the central portion were necrotic and autolysing. The capsule and its branches were composed of tissue which closely copied, in structure and, in some places, in arrangement, the cortex of the normal suprarenal. Where the capsule was thick, especially in the case of the smaller tumour, the resemblance to the zona glomerulosa was very striking. The interlobular septa were composed of sheets, columns, and masses of cells of the same appearance as, and directly

### PLATE III.

(*a*) Fig. 1. (*b*)

*External genital organs.*

L.—Atrophied labia.

C.—Hypertrophied clitoris.

G.—Groove in clitoris.
U.—Urethral orifice.

Fig. 2.

*Internal genital organs.*

P.—Prostate. C.—Clitoris with probe in urethra. U.—Uterus. O.—Ovary

Fig. 3

*Micro-photograph of section of prostate.*

Note resemblance to gland in "senile hypertrophy" in males.

### PLATE IV.

FIG. 4.—*A very low-power view of the surface of the tumour.*

C.—Capsule of supra-renal cortex.
D.—Sarcomatous-looking tissue embedded in fat.

FIG. 5.—*Higher-power view of central part of tumour.*

A string of supra-renal cortical cells (S) traverses the section. Masses of small round cells in fat invest them.

(*a*)

Shows the resemblance in structure and arrangement to the zona fasciculata.

(*b*)

A string of similar cells in centre of tumour.

(*c*) High-power view of cells showing vacuolation where lipoid has been dissolved out, and pigment granules in cytoplasm.

FIG. 6.—*Showing the structure of the cortical tissue*

continuous with, those forming the capsule. In the larger septa these cells were fifteen to twenty cells thick ; in the smallest they were composed of similar cells strung out in single file. The cells were large, average diameter 12$\mu$. in apposition, and spherical to polyhedral in shape. Their abundant, strongly acidophile cytoplasm was crowded with lipoid droplets staining, but indifferently, with Scharlach R, and dusted over with brownish-yellow pigment granules. Their nuclei were central, ovoid and relatively small, sharply but not deeply staining, and showing a simple, finely-dotted reticulum, sometimes with one or two rather small karyosomes.

The cells forming this suprarenal-like tissue were fairly true to type. They did not form any structure resembling gland tubules and had no tendency to do so. The connective tissue was scanty and the vessels primitive, being either sinusoids lined by tumour cells or capillaries with single cell walls.

The most remarkable constituent of the tumours was the small cell tissue, which under low powers, in parts where fat was absent, gave the impression of a round-celled sarcoma, but in other sections, where fat was present, closely resembled bone marrow. The latter resemblance was very striking in the majority of sections, for this sarcomatous-looking tissue had apparently spread into the fasciculi of suprarenal cells, and shaving off a cell here and there, had isolated it. Such cells, swollen and degenerate, often showing much nuclear distortion and irregularity, were seen lying surrounded by round cells with deeply-staining nuclei scattered in adipose tissue, the whole producing a very fair imitation of megakaryocytes and the elements of bone marrow. That this resemblance was probably only superficial was suggested by finding in these isolated cells in frozen section the same lipoid droplets and pigment granules as were found in the cells composing the capsule and its interlobular septa.

The nature of this round-celled tissue is obscure. Some possibilities are :—

1. That it is bone marrow derived from the tissue of the corresponding body segment, misplaced during the development of the suprarenal cortex from the Wolffian ridge.

2. That it represents the medulla of the glands or is a new growth (neuroblastoma) of this tissue.

3. That it is sarcomatous and derived from the gland stroma.

Many attempts were made to stain specific granules in the cells to support the first hypothesis and to demonstrate neuro-fibrils in support of the second, but without success. The giant cells in this tissue are probably not megakaryocytes because of their heavy load of lipoid. It is unlikely that these cells represent a neuro-blastomatous growth because of the absence of rosette formation, neuro-fibrils and metastases. The exact nature of this constituent is thus uncertain, but we are inclined to regard it as sarcomatous, chiefly because of the lack of arrangement, the evidence of vigorous growth and absence of differentiation of its cells. This conclusion is supported by the history, *i.e.* recent rapid enlargement of the tumour sufficient to produce acute obstruction and the presence in both tumours of recent hemorrhage.

### Summary of Case Report.

A woman of 70, who had been catheterised several times during the last few months of her life for retention of urine, was admitted to hospital for intestinal obstruction.

A large tumour mass was found replacing each suprarenal gland. The tumours were due to bilateral cortical hyperplasia of the suprarenals with diffuse fatty infiltration: their stroma was probably sarcomatous.

Her secondary sex characters approximated to the male type, the voice, physique and hirsuties being masculine. Considerable hypertrophy of the clitoris was present. The uterus, ovaries and vagina were rudimentary.

The prostate was enlarged, its "middle lobe" sufficiently so to cause a degree of retention only relieved by catheterisation.

No tissue resembling the male testicle was found in the pelvis.

### Summary of Pathological Examination of Suprarenal Tumours.

A greasy tumour mass replaced each suprarenal gland, that on the left side weighing several pounds.

Neither tumour was infiltrating the surrounding tissues. An intact capsule, very closely resembling the suprarenal cortex in histological structure and arrangement, enclosed each tumour, and branches from this, of similar structure, formed a wide-meshed, irregular network in its interior.

The meshes of the network were filled by hemorrhagic fat. Lying deep to the capsule was a cellular layer equal to it in thickness, and composed of small round cells infiltrating the underlying fat. Similar cells invested the branches of the capsule in the interior of the tumour.

*Pathological Diagnosis.*—Bilateral cortical hyperplasia of the suprarenal glands with sarcomatous change in their stroma.

### Comments.

There is little doubt that in this case we are concerned entirely with an abnormality of internal secretion, and it may be taken as a general rule that the more closely the suprarenal hyperplasia or tumour formation copies the mother tissue the more developed will be the symptoms of virilism. In our case the resemblance was close, when allowance is made for distortion, hemorrhage and necrosis.

There are several points of interest in the interrelation between the hormones associated with sex characters, some of which our case clearly illustrates. Firstly, it is probable that the hormones of one sex do not neutralise those of the other: both may act together. Secondly, the somatic cells of the body have the potentialities of both sexes. Thirdly, the close relation which exists between the cortex of the suprarenal and the gonads is supported by their origin from a common anlage, and by the fact that in rabbits who survive extirpation of both suprarenals for a sufficient time the interstitial cells of the ovary show hyperplasia ; and lastly, gonadectomy, pregnancy and ovulation are all accompanied by hyperplasia of the suprarenal cortex.

The literature, especially the contributions of Bulloch and Sequeira[1] and Glynn[2, 3] contain many references to virilism of suprarenal origin, and there are two cases[4, 5] of virilism in females in which, following removal of a suprarenal tumour, the pronounced male characters disappeared, partially in one, completely in the other. In another[6] removal of an ovarian tumour, possibly a hyperplastic suprarenal rest, was followed by a similar result.

We are much indebted to Miss C. I. Cooke, B.Sc., of the Pathological Department of the General Hospital for valuable technical assistance.

REFERENCES.

1 Bulloch and Sequeira, *Tr. Path. Soc.*, 1905, lvi. 189.

2 E. E. Glynn, *Quart. J. Med.*, 1912, v. 157.

3 E. E. Glynn, *Jour. of Obst. and Gyn. of Brit. Emp.*, Spring, 1921.

4 Gordon Holmes, *Quart. J. Med.*, 70, January, 1925.

5 A. Collet, *Amer. J. Child. Dis.*, 1924, 27, 208–218.

6 E. Bovin, *Nord. Med. Arkiv.*, Stockholm, 1908, 3° F., viii., afd. I., No. 15, 1–35.

## Figures and Tables

**Fig. 1. f1:**
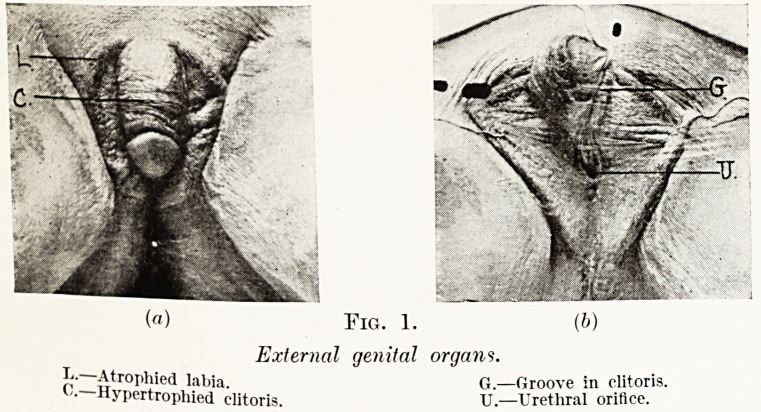


**Fig. 2. f2:**
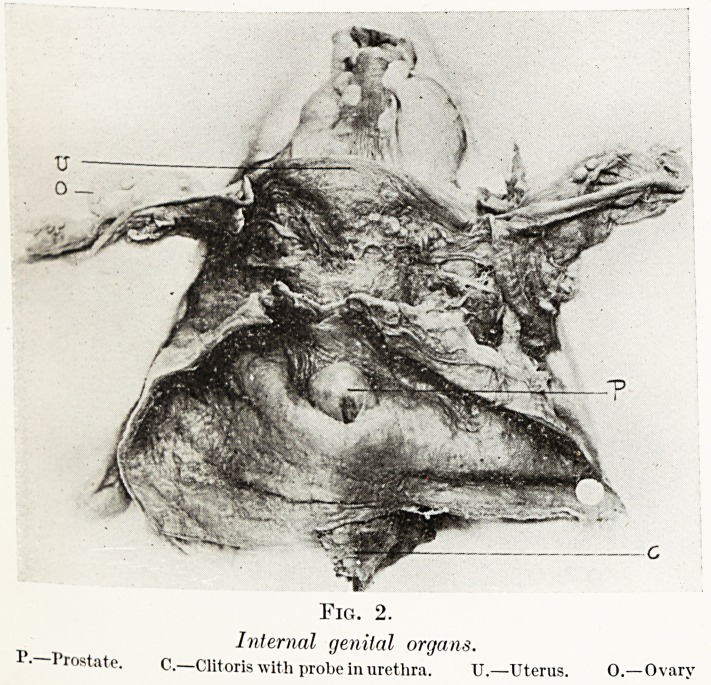


**Fig. 3 f3:**
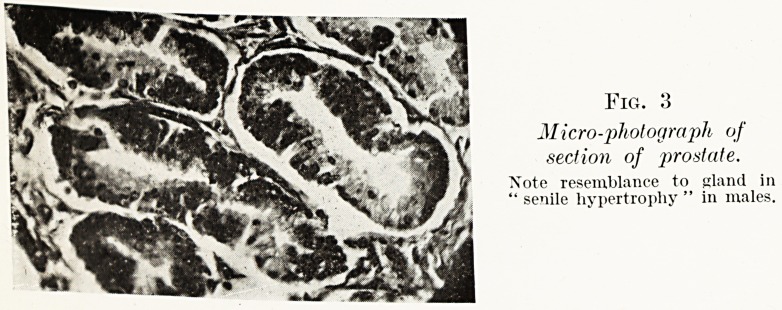


**Fig. 4. f4:**
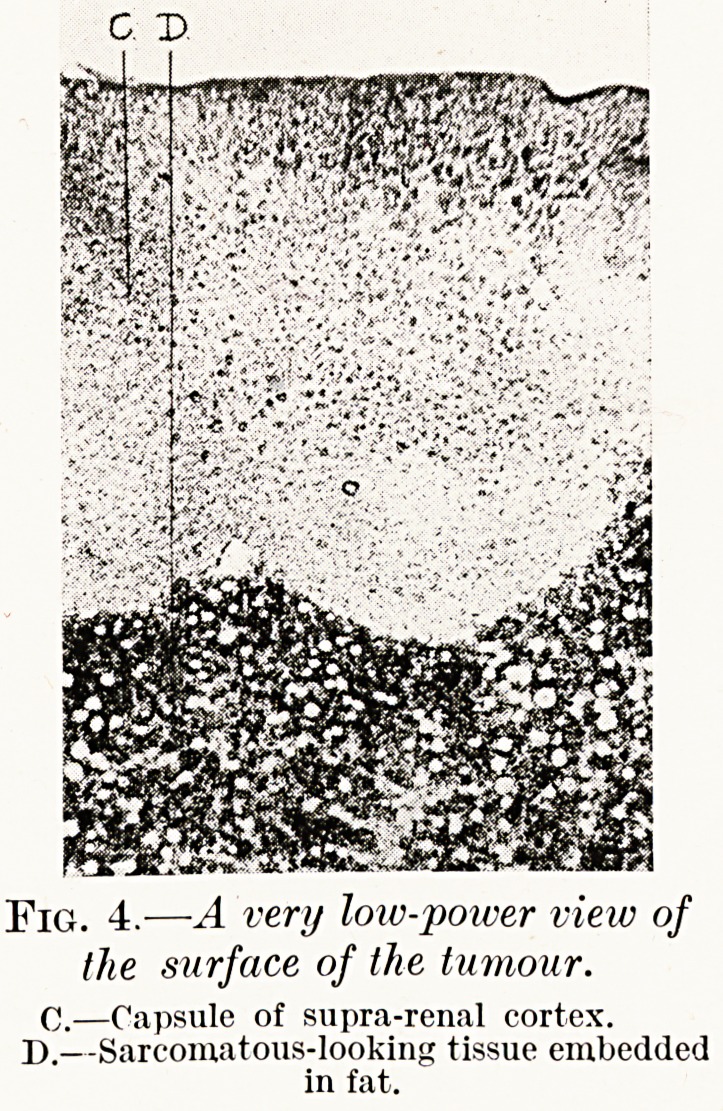


**Fig. 5. f5:**
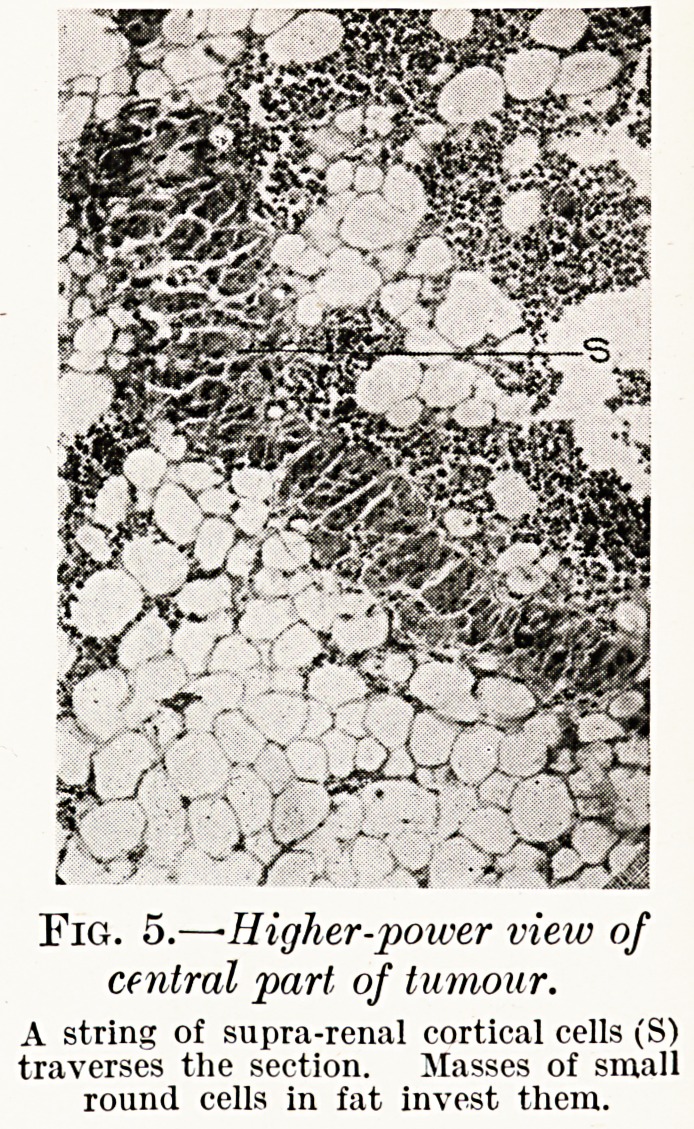


**Fig. 6. f6:**